# Antioxidant activity of banana flesh and antiproliferative effect on breast and pancreatic cancer cells

**DOI:** 10.1002/fsn3.2702

**Published:** 2022-01-26

**Authors:** Dae Kyeong Kim, Meran Keshawa Ediriweera, Munkhtugs Davaatseren, Ho Bong Hyun, Somi Kim Cho

**Affiliations:** ^1^ Interdisciplinary Graduate Program in Advanced Convergence Technology and Science Jeju National University Jeju South Korea; ^2^ Subtropical/Tropical Organism Gene Bank Jeju National University Jeju South Korea; ^3^ Biodiversity Research Institute Jeju Technopark Jeju South Korea; ^4^ Department of Biotechnology College of Applied Life Sciences Jeju National University Jeju South Korea; ^5^ Present address: Department of Biochemistry and Molecular Biology Faculty of Medicine University of Colombo Colombo Sri Lanka

**Keywords:** anticancer activity, apoptosis, banana, breast cancer, pancreatic cancer

## Abstract

Bananas, one of the most widely consumed fruits worldwide, are a rich source of valuable phytochemicals. In this study, the antioxidant and the anticancer potential of banana flesh was investigated. Of the four kinds of banana flesh extracts, the hexane extract (HE) had the highest total polyphenol content (2.54 ± 0.60 mg GAE/g) and total flavonoid content (1.69 ± 0.34 mg RE/g), followed by the chloroform fraction, total ethanol extract, and ethanol fraction. HE was found to exert a strong radical scavenging activity on 2,2‐diphenyl‐1‐picrylhydrazyl (DPPH•) and 2,2′‐azino‐bis(3‐ethylbenzothiazoline‐6‐sulfonicacid) (ABTS•) free radicals. According to the IC_50_ values in various cancer cell lines, HE was found to possess the greatest cell growth inhibitory potential in human pancreatic cancer PANC‐1 cells and human triple‐negative breast cancer MDA‐MB‐231 cells. HE induced apoptosis in PANC‐1 and MDA‐MB‐231 cells, as evidenced by the appearance of condensation of chromatin, proteolytic activation of caspase‐3 and 7, and increase in the level of the cleaved form of poly (ADP‐ribose) polymerase protein. Gas chromatography mass spectrometry (GC‐MS) analysis of HE identified several anticancer compounds including palmitic acid, linoleic acid, oleic acid, campesterol, stigmasterol, and γ‐sitosterol, supporting the anticancer potential of HE. Our investigation provides a rationale for the use of banana flesh to minimize the risk of cancer‐like diseases.

## Graphical abstract

The antioxidant and anti‐cancer potential of banana flesh was investigated. The hexane extract of banana flesh (HE) showed strong antioxidant activity. HE was found to possess the cell growth inhibitory potential in human pancreatic cancer PANC‐1 cells and human triple‐negative breast cancer MDA‐MB‐231 cells. HE induced apoptosis in PANC‐1 and MDA‐MB‐231 cells, as evidenced by the appearance of condensation of chromatin, proteolytic activation of caspase‐3 and 7, and increase in the level of the cleaved form of poly(ADP‐ribose) polymerase protein.

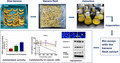

## 1 INTRODUCTION

Fruits are a rich source of vitamins and sugars, along with diverse phytochemical contents, and have been linked to reducing the risk of major chronic degenerative diseases including cancer.

Banana, one of the widely consumed fruits worldwide, belongs to the genus *Musa* in the family Musaceae (Singh et al., 2016). The genus *Musa* contains approximately 70 species, with more than 300–500 different varieties (Häkkinen, 2009; Maduwanthi & Marapana, 2019). World production of banana is approximately 116 million tons and India is the country with the greatest banana production, at approximately 30 million tons per year (FAO, 2019).

Banana fruit peel and flesh are rich sources of valuable phytochemicals, including polyphenols, flavonoids, fatty acids, carotenoids, phytosterols, amines (Ajijolakewu et al., 2021; Mathew & Negi, 2017; Qamar & Shaikh, 2018; Someya et al., 2002; Vilela et al., 2014). Among these phytochemicals, polyphenols, fatty acids, and phytosterols are abundant in banana. Catechin, epicatechin, gallic acid, cinnamic acid, chlorogenic acid, and protocatechuic acid are examples of major polyphenolics found in banana, while phytosterols such as stigmasterol, cycloeucalenone, beta‐sitosterol, and cycloartenol are examples of banana phytosterols. (Pereira & Maraschin, 2015).

Banana flesh has been reported to possess promising bioactive antioxidant compounds, which contribute to defense mechanisms against free radicals, thereby mediating oxidative damage in cells (Qamar & Shaikh, 2018). Phenolic compounds, vitamins, catecholamines, and carotenoids present in banana are responsible for this antioxidant potential (Vu et al., 2019). A recent study demonstrated that acetone, ethanol, and aqueous extracts of two banana varieties (*Musa sinensis* L. and *Musa paradisiaca* L.) collected from the Eastern Cape Province of South Africa have strong antioxidant effects (Oyeyinka & Afolayan, 2020). Another study reported that two polyphenolics found in banana, catechin and quercetin, strongly contributed to the antioxidant activities of banana varieties collected in Brazil. Sulaiman et al. (2011) assessed the antioxidant potentials of eight banana varieties grown in Malaysia and reported that the antioxidant properties of those banana varieties were correlated with the phenolic contents of banana flesh and peel. Vijayakumar et al. (2008) demonstrated that flavonoids have a significant effect on the antioxidant activity of bananas collected in India. In another investigation, aqueous extracts prepared from three Nigerian banana varieties exhibited antioxidant properties (Adedayo et al., 2016).

Although several studies have reported the antioxidant potentials of various banana varieties, studies assessing the potential anticancer effects of bananas are extremely limited. Therefore, an investigation was conducted to assess the potential antiproliferative and apoptotic effects of the flesh of bananas grown on Jeju Island, South Korea, in human pancreatic and breast cancer cells.

## 2 MATERIALS AND METHODS

### 2.1 Collection of plant material and extraction

The banana samples (*Musa cavendishii* Lamb.) cv. Dwarf Cavendish were obtained from Yujinfang (Seogwipo, Jeju, South Korea). The taxonomic identification of the collected banana sample has been reported in a previous study (Kim, 1988). A voucher specimen (number SKC. 200427) was deposited in the laboratory of Professor Somi Kim Cho at the College of Applied Life Sciences, Jeju National University, South Korea. After ripening, banana fruit peel and flesh were carefully separated. The fruit flesh was then cut into small pieces and freeze‐dried. The freeze‐dried material (300 g of freeze‐dried flesh subjected to three rounds of sonication in 1 L of each solvent) was extracted with 70% ethanol (1 L ethanol for three rounds of sonication) to obtain total ethanol extract (TE). Another portion (300 g) of freeze‐dried banana flesh was then extracted sequentially in hexane, chloroform, and 70% ethanol to obtain hexane extract (HE), chloroform fraction (CF), and ethanol fraction (EF). All resulting extracts were evaporated under reduced pressure and stored at −20°C until use.

### 2.2 Determination of total polyphenol and flavonoid contents

The total polyphenol content (TPC) and total flavonoid content (TFC) were determined as described previously (Singleton et al., 1999). To measure TPC, 1375 µl of distilled water and 125 μl of flesh extract (8 mg/ml in DMSO) were mixed with 500 μl of Folin–Ciocalteu reagent and incubated for 10 min. Following incubation, 1 ml of 10% Na_2_CO_3_ was mixed with the reaction mixture and incubated for a further 30 min in the dark. After incubation, the absorbance was measured using a microplate reader at 700 nm. The TPC was expressed as milligrams of gallic acid equivalent (GAE) per gram of banana extract. To estimate TFC, reaction mixtures comprised of 40 μl of flesh extract (8 mg/ml in DMSO), 80 μl of distilled water, and 6 μl of 5% NaNO_2_ were prepared. Following 5 min of incubation, the reaction mixtures were mixed with 12 μl of 10% AlCl_3_ and incubated for 6 min at room temperature. Then, 40 μl of 1 N NaOH was added to each reaction mixture and the absorbance was recorded at 510 nm. TFC of banana extracts was expressed as milligrams of rutin equivalent (RE) per gram of extract.

### 2.3 Analysis of the antioxidant activities of banana extracts

#### 2.3.1 Radical scavenging activity of 2,2‐diphenyl‐1‐picrylhydrazyl (DPPH)

The radical scavenging activity of DPPH in banana flesh extracts was determined as previously described, with minor modifications (Ryu et al., 2020). Prior to the assay, 160 μl of reaction mixtures containing freshly prepared DPPH solution (200 μM) and 40 µl (5, 10, and 20 mg/ml in DMSO) of each extract were mixed, and incubated at 37°C for 30 min. Following incubation, the absorbance was measured using a microplate reader at 517 nm. Catechin was used as the positive control. To calculate the percentage radical scavenging activity, the following formula was used: (absorbance of controls – absorbance of treatment group) ÷ (absorbance of controls) × 100%. Half maximal effective concentration (EC_50_) values for each banana extract were calculated using GraphPad Prism 7.0 software (GraphPad Software, Inc.).

#### 2.3.2 Radical scavenging activity of 2,2’‐azino‐bis(3‐ethylbenzothiazoline‐6‐sulfonic acid) (ABTS)

An ABTS radical scavenging activity assay was conducted as previously described (Ryu et al., 2020). Prior to the assay, ABTS stock solution (7 mM ABTS in 2.45 mM potassium persulfate) was prepared and incubated at room temperature for 20 h. Following incubation, 900 µl of the ABTS solution was mixed with 100 µl (5, 10, and 20 mg/ml in DMSO) of banana extract, incubated for 2 min, and the absorbance was recorded using a microplate reader at 734 nm. The percentage radical scavenging activity of each extract was calculated using the following formula: (absorbance of controls − of treatment group) ÷ (absorbance of controls) × 100%. EC_50_ values for each banana extract were calculated using GraphPad Prism 7.0 software (GraphPad Software, Inc.).

### 2.4 Cell culture

Human triple‐negative breast cancer MDA‐MB‐231 cells, human pancreatic cancer PANC‐1 cells, human hepatoblastoma HepG2 cells, normal mammary epithelial MCF‐10A cells, and murine macrophage RAW 264.7 cells were cultured using the media recommended by the American Type Culture Collection (ATCC) and maintained at 37°C under an atmosphere of 5% CO_2_. Radio‐resistant breast cancer MDA‐MB‐231/IR and stem‐like breast cancer MCF‐7/SC cells were cultured in Dulbecco's modified Eagle medium (DMEM) supplemented with 10% fetal bovine serum (FBS) and maintained at 37°C under an atmosphere of 5% CO_2_.

### 2.5 3‐(4,5‐dimethylthiazol‐2‐yl)‐2,5‐diphenyltetrazolium bromide (MTT) assay

The MTT assay was conducted as previously described (Nguyen et al., 2020). Briefly, murine macrophage RAW 264.7 cells, human triple‐negative breast cancer MDA‐MB‐231 cells, and radio‐resistant triple‐negative breast cancer MDA‐MB‐231/IR established and characterized as previously described (Koh et al., 2019), human pancreatic cancer PANC‐1 cells, human hepatoblastoma HepG2 cells, breast cancer stem cell line MCF‐7SC established and characterized as previously described (To et al., 2020; Van Phuc et al., 2011), and normal mammary epithelial MCF‐10A cells (5000 cells/well) were cultured in 96‐well plates and incubated for 24 h. Following incubation, the cells were exposed to various banana flesh extracts and incubated for a further 48 h. Then, the cells were washed with phosphate‐buffered saline (PBS), 20 μl of 1 mg/ml MTT solution was added to each well, and the cells were incubated at 37°C for 4 h. Following incubation, 200 μl of dimethyl sulfoxide (DMSO) was added to each well to solubilize formazan and the culture plates were shaken for 1 h at room temperature. The absorbance was recorded at 570 nm using a microplate reader. The percentage of cell viability was calculated using the formula: (absorbance of untreated controls – absorbance of treatments) ÷ (absorbance of untreated controls) × 100% and half‐maximal inhibitory concentration (IC_50_) values for each extract were calculated using GraphPad Prism 7.0 software.

### 2.6 Nitric oxide (NO) production assay

RAW 264.7 cells were seeded into 96‐well cell culture plates and incubated for 24 h. Following incubation, cells were pretreated with banana extracts in a concentration‐dependent manner for 30 min, and then exposed to 1 μg/ml of lipopolysaccharide (LPS) for 24 h. Next, 100 μl of sodium nitrite standard solutions and culture media collected after 24 h treatment was added into new 96‐well cell culture plates, and mixed with 100 μl of Griess reagent. The mixture was incubated for 10 min at room temperature and optical density was measured at 550 nm using a microplate reader.

### 2.7 Colony formation assay

PANC‐1 (400 cells/dish) and MDA‐MB‐231 (200 cells/dish) cells were seeded into 60‐cm diameter cell culture dishes and incubated for 24 h. Then, the cells were exposed to various concentrations of HE for 10 days. The medium was removed from culture dishes, and the cells were washed with PBS, fixed with 4% formaldehyde for 15 min, followed by staining with 0.5% crystal violet for 30 min. The stained cell colonies were washed briefly with PBS and air‐dried for 4 h. The photographic images of colonies were taken and numbers of colonies were counted with ImageJ software (version 1.48; National Institutes of Health, Bethesda, MD, USA).

### 2.8 Western blot analysis

Following exposure to HE at the indicated concentrations for 48 h, lysates of PANC‐1 and MDA‐MB‐231 cells were prepared using radioimmunoprecipitation assay (RIPA) buffer (RIPA Lysis and Extraction Buffer, cat#89900, Thermo Fisher Scientific). The protein concentrations of cell lysates were quantified using the bicinchoninic acid (BCA) assay kit according to the manufacturer's manuals (Pierce™ BCA Protein Assay Kit, cat#23225, Thermo Fisher Scientific). Following quantification, equal amounts of proteins were loaded into 10% sodium dodecyl‐sulfate polyacrylamide gel electrophoresis (SDS‐PAGE) gel and transferred electrophoretically onto polyvinylidene difluoride (PVDF) membranes. The membranes were then blocked with 5% nonfat‐dried skim milk for 12 h at 4°C and exposed to various primary antibodies for 24 h at 4°C. Primary and secondary antibodies were diluted as recommended by the suppliers (Cell Signaling Technology). Enhanced Chemiluminescent (ECL) Reagent Kit (BS ECL‐Plus kit, cat#W6002, Biosesang, Inc.) was used to develop protein bands via Chemidoc (ChemiDoc™ MP Imaging System, cat#12003154, Bio‐Rad Laboratories). Developed bands were quantified using ImageJ software (version 1.48; National Institutes of Health, Bethesda, MD, USA).

### 2.9 Annexin V‐fluorescein isothiocyanate (FITC) and propidium iodide (PI) staining

The apoptotic effects of HE were investigated using the Annexin V‐FITC Apoptosis Detection Kit (BD Biosciences) according to the manufacturer's instructions. Briefly, PANC‐1 (5 × 10^4^/well) and MDA‐MB‐231 cells (1 × 10^4^/well) were seeded in 6‐well cell culture plates and incubated for 24 h. The cells were then exposed to HE at the indicated doses for 48 h. Next, the cells were collected as pellets, re‐suspended in 100 μl binding buffer, and stained with 2.5 μl PI and 2.5 μl annexin V for 15 min. Thereafter, the cells were analyzed with a flow cytometer (BD Biosciences) at the Bio‐Health Materials Core Facility of Jeju National University.

### 2.10 Hoechst 33342 staining

PANC‐1 (5 × 10^4^/well) and MDA‐MB‐231 cells (1 × 10^4^/well) were seeded into cell culture dishes and incubated for 24 h. Following incubation, cells were treated with HE for 48 h. Then, the cells were fixed with 4% formaldehyde, and stained with Hoechst 33342 solution (0.01 mg/ml) for 10 min. Stained PANC‐1 and MDA‐MB‐231 cells were observed under a fluorescence microscope (×100) (IX73; Olympus Corporation).

### 2.11 Gas chromatography mass spectrometry (GC‐MS) analysis

GC‐MS analysis of HE was carried out using the Agilent 7890A (GC)‐Agilent 5975C inert MSD with a triple‐axis detector from Agilent Technologies. The J&W CP‐Sil‐8 GC column (30 m, 0.25 mm, and 0.25 µm) was used for GC separation. The injected sample volume was 1 µl (5 mg/ml dissolved in methanol) and helium was used as the carrier gas at a constant flow rate of 1 ml/min. Samples were injected in split mode (1:20). The GC oven temperature was programmed to rise from 50°C (held for 1 min) to 120°C at 10°C/min and, finally, to 280°C at 5°C/min (held for 2 min). The total run time was 65 min. The mass spectra of compounds present in HE were matched with the W9N08 Wiley library ver. 9.0 at a similarity cut‐off of 85%.

### 2.12 Statistical analysis

All experiments in the present study were conducted in triplicate (*n* = 3) and the results are expressed as the mean ± standard deviation (SD) of the three independent experiments. GraphPad Prism version 7 (GraphPad Software, Inc.) software was used for statistical analysis. For group comparisons, one‐way analysis of variance (ANOVA) was used with Dunnett's post hoc test. *p* < .05 was used as the threshold for statistical significance.

## 3 RESULTS AND DISCUSSION

### 3.1 Total polyphenol content (TPC) and total flavonoid content (TFC)

**FIGURE 1:**
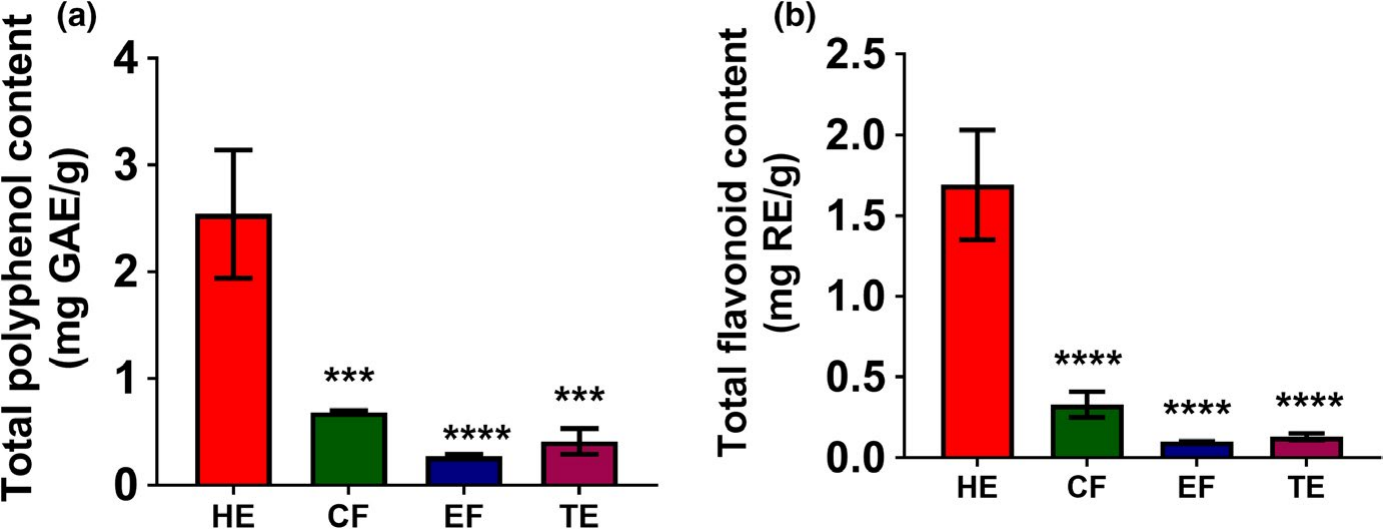
TPC and TFC of four banana flesh extracts, hexane extract (HE), chloroform fraction (CF), ethanol fraction (EF), and total ethanol extract (TE). Statistical comparison among groups was carried out using one‐way ANOVA with Dunnett's post hoc test. **p < .01, ***p < .001, and ****p < .0001 (*n* = 3)

Phenolic and flavonoid compounds, which are abundant secondary metabolites in many fruits and vegetables, exhibit a wide range of biological activities (Kim et al., 2021). Due to their distinct biological activities, phenolics and flavonoids are attractive food ingredients in the field of food science and technology research (Gutiérrez‐Grijalva et al., 2018). Previous studies have reported that phenolics and flavonoids have a direct impact on the antioxidant potential of fruits and vegetables (Gutiérrez‐Grijalva et al., 2018; Kim et al., 2021). Extracts of fruits such as apple, mango, plum, strawberry, gooseberry, blackcurrant, mulberry, and apricot have been reported to show antioxidant effects that might be related to the phenolic and flavonoid contents of the extracts (Russell et al., 2009; Sultana & Anwar, 2008). The TPC and TFC of four extracts, total ethanol extract (TE), HE, chloroform fraction (CF), and ethanol fraction (EF) of banana flesh are shown in Figure 1. Of the four extracts, the HE of banana flesh had the highest TPC (2.54 ± 0.60 mg GAE/g) and TFC (1.69 ± 0.34 mg RE/g), followed by the CF and TE.

TE had higher TPC and TFC values than the EF obtained through sequential extraction in a significant manner (*p* < .0001). However, TE had lower TPC and TFC than the CF and HE (Figure 1). Sulaiman et al. (2011) reported TPC and TFC values of the hexane, chloroform, and methanol extracts of Malaysian banana flesh extracts obtained through sequential extraction. According to their findings, the chloroform extract of banana flesh of the Awak variety obtained following sequential extractions displayed the highest TPC (23.42 ± 1.22 mg GAE/g dry weight). The reason for this deviation might be differences in the banana varieties and extraction methods used in that particular experiment, as variety and extraction method both show significant effects on the TPC and TFC of banana extracts (Oyeyinka & Afolayan, 2020; Borges et al., 2020; Sulaiman et al., 2011; Vijayakumar et al., 2008; Adedayo et al., 2016).

### 3.2 Antioxidant activities

**FIGURE 2:**
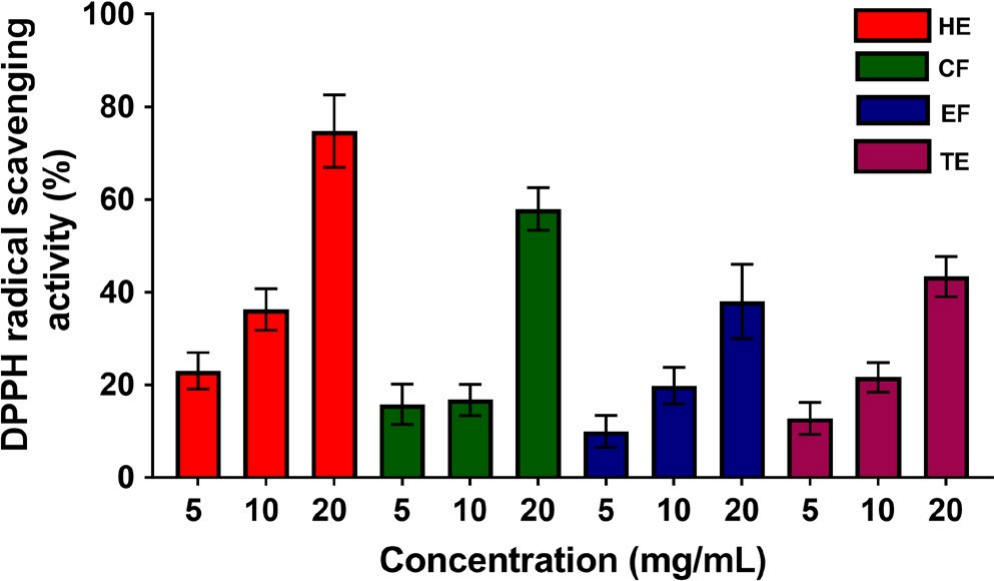
DPPH scavenging ability of four banana flesh extracts, hexane extract (HE), chloroform fraction (CF), ethanol fraction (EF), and total ethanol extract (TE) (*n* = 3)

**FIGURE 3:**
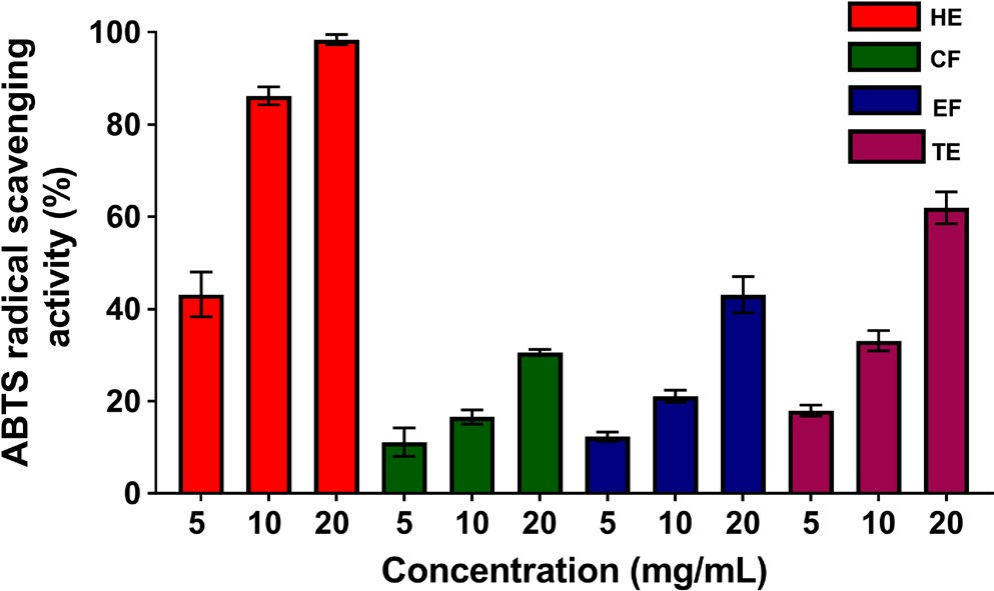
ABTS· scavenging ability of four banana flesh extracts four banana flesh extracts, hexane extract (HE), chloroform fraction (CF), ethanol fraction (EF), and total ethanol extract (TE) (*n* = 3)

Several studies have demonstrated the antioxidant activities of various fruit and vegetable extracts (Ediriweera et al., 2017; Kamiloglu et al., 2016; Kevers et al., 2007). To obtain a clear profile of the antioxidant potential of banana flesh extracts, DPPH and ABTS radical scavenging assays were performed, due to these assays having been widely applied to assess the antioxidant effects of numerous fruit and vegetable extracts (Kim et al., 2021). DPPH· is a stable free radical that is reduced to colorless DPPH upon exposure to antioxidants (Mishra et al., 2012). As shown in Figure 2, the DPPH· scavenging abilities of all four banana extracts increased in a concentration‐dependent manner. The highest dose tested (20 mg/ml) displayed peak DPPH· scavenging activity for all four extracts in a significant manner (Figure 2). HE exhibited the greatest DPPH· scavenging ability compared with that of the other extracts. The EC_50_ values obtained from DPPH· scavenging activities of HE, CF, EF, and TE were 12.11 ± 0.70, 22.38 ± 1.84, 37.66 ± 9.73, and 28.91 ± 3.09 mg/ml, respectively. ABTS· can also be reduced to colorless ABTS upon exposure to antioxidants (Miller & Rice‐Evans, 1997). Similar to the results of the DPPH· assay, all four extracts showed increasing ABTS· scavenging activity in a concentration‐dependent manner, and the highest dose tested (20 mg/ml) displayed peak ABTS· scavenging activity for all four extracts in a significant manner (Figure 3). Similar to DPPH results, the HE had shown the strongest ABTS· scavenging activity (>90% at 20 mg/ml) compared with that of the other extracts. The EC_50_ values obtained from ABTS· scavenging activities of HE, CF, EF, and TE were 3.44 ± 0.33, 46.16 ± 2.60, 30.86 ± 3.79, and 16.85 ± 1.75 mg/ml, respectively. Consistent with our experiments, Adedayo et al. (2016) reported that aqueous extracts of banana flesh extracts of three Nigerian varieties have free radical scavenging effects at concentrations ranging from 5 to 25 mg/ml. Furthermore, Sulaiman et al. (2011) demonstrated that Malaysian banana flesh extracts obtained through sequential extraction with hexane, chloroform, and methanol exhibit strong free radical scavenging effects. The HE of flesh of Kapas variety (1.92 Trolox equivalent/g dry weight of extract), the chloroform extract of Nipah variety (2.80 Trolox equivalent/g dry weight of extract), and the methanol extract of Berangan variety (2.15 Trolox equivalent /g dry weight of extract) obtained following sequential extractions displayed highest free radical scavenging activities (Sulaiman et al., 2011). In addition, Oyeyinka and Afolayan (2020) reported the free radical scavenging potentials of acetone and ethanol extracts of *Musa sinensis* and *Musa paradisiaca* (South African) varieties showed peak antioxidant effects at the highest concentration (0.01 mg/ml) tested (Oyeyinka & Afolayan, 2020), which supports that the antioxidant activity of banana flesh extract could differ depending on extraction methods and solvents used in extractions.

### 3.3 Inhibition of NO production in LPS‐stimulated RAW 264.7 cells by hexane extract of banana fruit flesh

**FIGURE 4:**
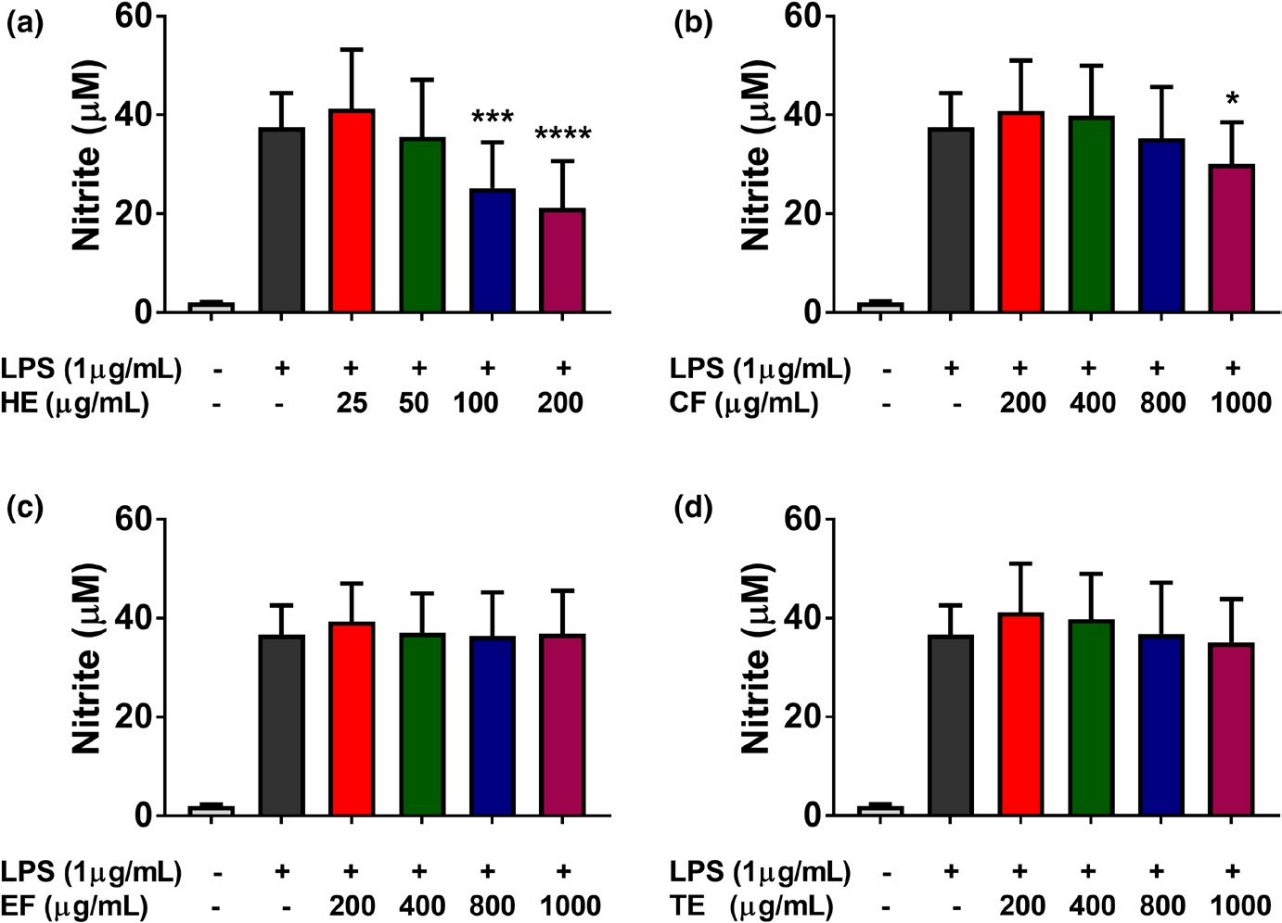
Effects of four banana flesh extracts, hexane extract (a), chloroform fraction (b), ethanol fraction (c), and total ethanol extract (d) on lipopolysaccharide (LPS)‐induced NO production in RAW 264.7 cells. Statistical comparison among groups was carried out using one‐way ANOVA with Dunnett's post hoc test. *p < .05, **p < .01, ***p < .001, ****p < .0001 vs. LPS (*n* = 3)

Normal levels of ROS and NO exert beneficial outcomes by contributing to cellular signaling pathways and maintaining physiological functions. However, overproduction of ROS and NO can cause irregularities in cellular homeostasis and damage to cellular components, leading to various diseases, including cancer. Therefore, natural remedies that control inflammatory responses through the mediation of ROS and NO levels are promising therapeutic strategies (Guzik et al., 2003; Swindle & Metcalfe, 2007). The LPS is a widely used activator of macrophages that mediates the production of pro‐inflammatory cytokines. The assessment of NO production inhibition in LPS‐induced RAW 264.7 cells is commonly used to identify plant extracts or secondary metabolites with anti‐inflammatory effects (Yoon et al., 2010). In the present study, banana extracts were evaluated for the inhibition of NO production in LPS‐induced RAW 264.7 cells (Figure 4). As shown in Figure 4, the nitrite concentration in RAW 264.7 cells increased following exposure to LPS. EF and TE did not reduce the LPS‐induced NO accumulation in RAW 264.7, whereas HE and CF significantly reduced at 100 and 1000 μg/ml, respectively. The cell viability assay showed that the inhibitory effects of these extracts were not due to cytotoxic effects (Figure S1). Collectively, these results indicate for the first time that banana flesh extracts may have the ability to mediate anti‐inflammatory responses through inhibition of NO accumulation in RAW 264.7 cells. Several lipophilic compounds identified in the HE, including stigmasterol and ethyl palmitate (Table 1), might be responsible for its anti‐inflammatory effect. Antwi et al. (2017) reported that stigmasterol can exert inhibitory effect on LPS‐induced potentially harmful innate immune responses. Khan et al. (2020) reported anti‐inflammatory mechanisms of stigmasterol in collagen‐induced arthritis which is attributed to the suppression of pro‐inflammatory cytokines. Vuan et al. (2019) demonstrated that phytosterols suppress phagocytosis and inhibit inflammatory responses in LPS‐treated RAW 264.7 macrophages. Saeed et al. (2012) demonstrated that ethyl palmitate can show anti‐inflammatory activity in several experimental inflammatory models in rats.

**TABLE 1:** Major lipophilic compounds in hexane extract (HE) tentatively identified through by GC–MS

NO	Compound	RT (min)	Area (%)^a^
1	Benzoic acid, 4‐ethoxy‐, ethyl ester	22.303	0.15 ± 0.12
2	Benzene, 1,2,3‐trimethoxy‐5‐(2‐propenyl)‐	22.833	0.17 ± 0.03
3	Phenol, 2,6‐dimethoxy‐4‐(2‐propenyl)‐	24.044	0.58 ± 0.05
4	Palmitic acid, methyl ester	31.02	0.35 ± 0.05
5	Palmitic acid	31.767	7.76 ± 0.73
6	Ethyl 9,12‐hexadecadienoate	32.014	0.25 ± 0.01
7	Ethyl palmitate	32.355	4.46 ± 0.18
8	Linoleic acid, methyl ester	34.261	0.20 ± 0.01
9	9,12,15‐Octadecatrienoic acid	34.373	0.21 ± 0.01
10	Linolenic acid	35.096	2.84 ± 0.38
11	Oleic Acid	35.155	1.94
12	Z‐6,17‐Octadecadien‐1‐ol acetate	35.467	2.97 ± 0.01
13	Ethyl linolenate	35.585	2.42 ± 0.03
14	Ethyl Oleate	35.685	0.77 ± 0.03
15	Butyl palmitate	35.902	0.15 ± 0.01
16	Ethyl stearate	36.032	0.16 ± 0.01
17	Ethyl linoleolate	39.879	0.09 ± 0.01
18	α‐Tocopherol	52.342	0.32 ± 0.03
19	Campesterol	55.389	2.53 ± 0.14
20	Stigmasterol	56.089	5.50 ± 0.06
21	γ‐Sitosterol	57.466	50.28 ± 0.93
22	(E)‐24‐Propylidene cholesterol	57.772	6.82 ± 0.17

a Data expressed as mean ± standard deviation (SD) (*n* = 3).

### 3.4 Effects of banana extracts on cell viability

**FIGURE 5:**
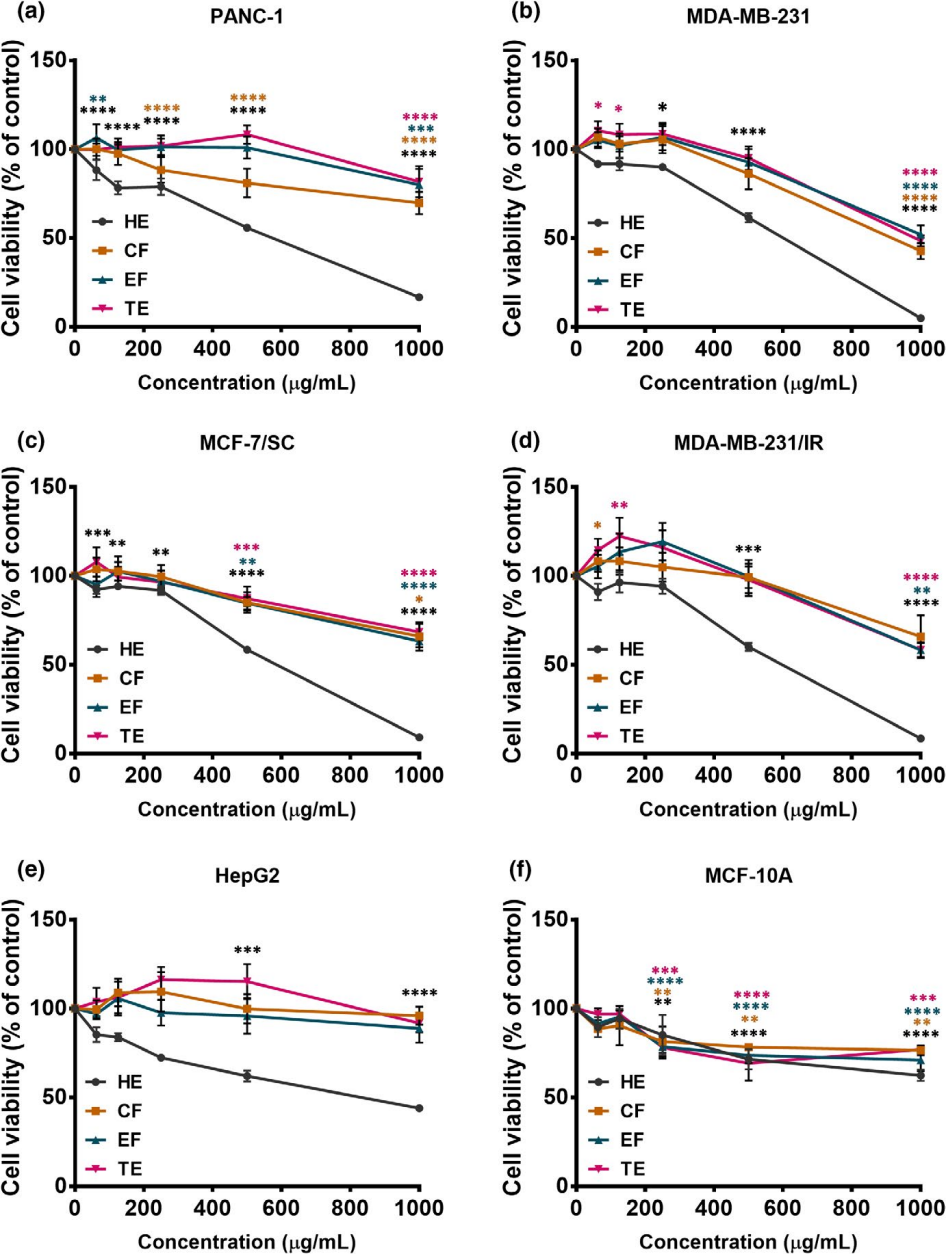
The antiproliferative potential of four banana flesh extracts, hexane extract (HE), chloroform fraction (CF), ethanol fraction (EF), and total ethanol extract (TE) in PANC‐1 cells (a), MDA‐MB‐231 cells (b), MCF‐7/SC cells (c), MDA‐MB‐231/IR cells (d), HepG2 cells (e), and MCF‐10A cells (f). The MTT assay was used to assess antiproliferative potential. Statistical comparison among groups was carried out using one‐way ANOVA with Dunnett's post hoc test. *p < .05, **p < .01, ***p < .001, and ****p < .0001 compared with the control group (*n* = 3)

**FIGURE 6:**
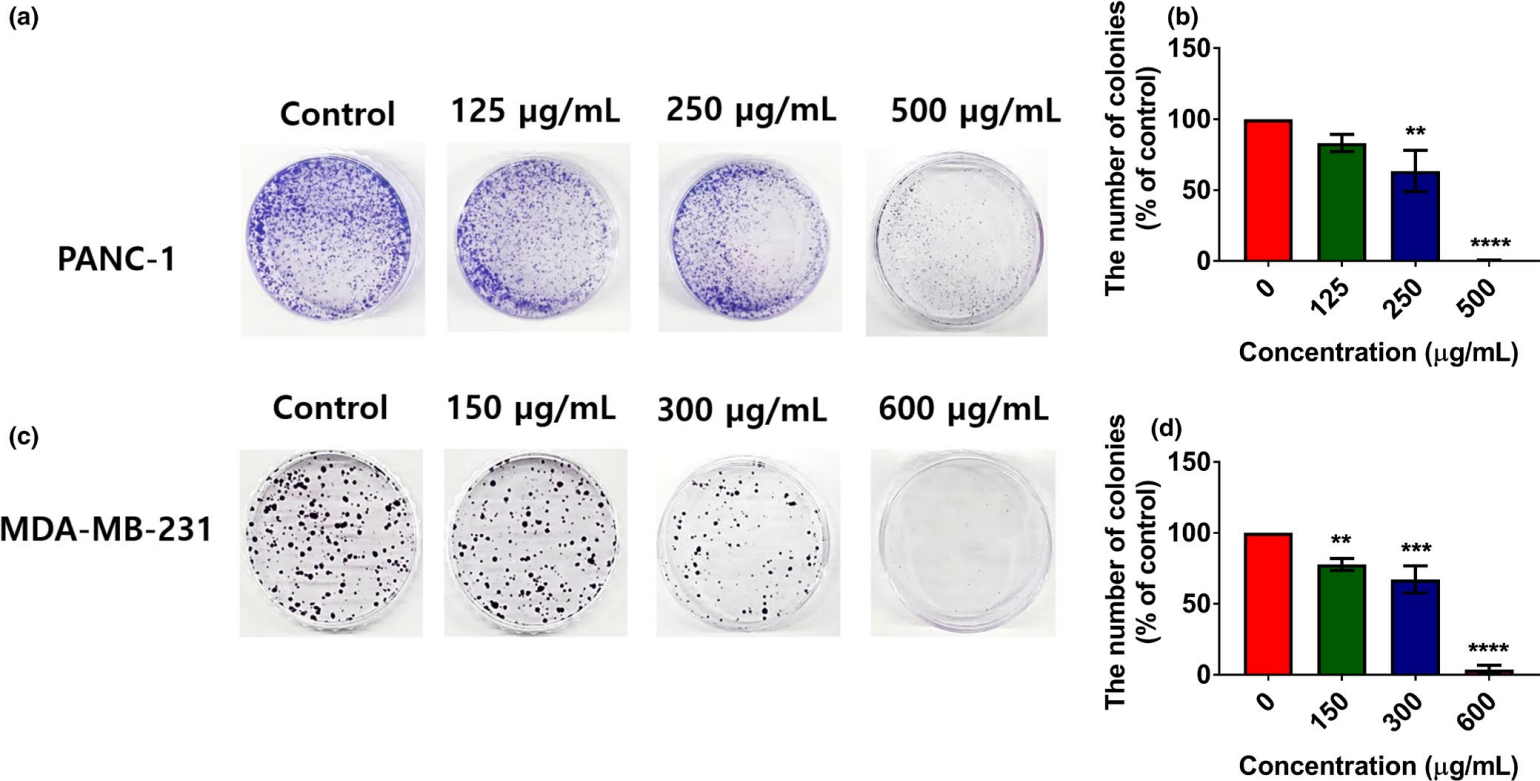
Effects hexane extract (HE) of banana flesh on the clonogenic ability of PANC‐1 (a, b) and MDA‐MB‐231 (c, d) cells assessed using the clonogenic assay following 10 days of exposure. Treatment dosages were selected based on the IC_50_ values of HE in PANC‐1 and MDA‐MB‐231 cells. Statistical comparison with the control group was carried out using one‐way ANOVA with Dunnett's post hoc test. **p < .01, ***p < .001, and ****p < .0001 (*n* = 3)

Uncontrolled cell proliferation is a hallmark of cancer cells. A number of studies have reported the efficacy of various plant extracts for inhibiting the proliferation of cancer cells (Ediriweera et al., 2019). The antiproliferative effects of banana extracts in human triple‐negative breast cancer MDA‐MB‐231 cells, MDA‐MB‐231/IR (radio‐resistant) cells, MCF‐7/SC (stem cell‐like) breast cancer cells, human pancreatic cancer PANC‐1 cells, human hepatoblastoma HepG2 cells, and the MCF10A human breast epithelial cells were assessed using the MTT assay after 48 h of incubation (Figure 5). Following exposure to banana extracts (HE, CF, EF, and TE), dose‐dependent inhibition of cancer cell proliferation was observed (Figure 5). The HE showed the strongest antiproliferative effects in all cancer cell lines tested (Figure 5). According to the IC_50_ values obtained for banana extracts in various cancer cell lines, HE was found to possess the greatest cell growth inhibitory potential in human pancreatic cancer PANC‐1 cells, with an IC_50_ value of 510.36 ± 60.69 µg/ml. The IC_50_ values obtained for HE were 592.26 ± 98.50, 616.1 ± 97.42, 638.2 ± 37.17, and 737.2 ± 110.62 µg/ml in MDA‐MB‐231, MCF‐7/SC, MDA‐MB‐231/IR, and HepG2 cells, respectively. HE showed a weaker cytotoxic effect in normal mammary epithelial cells, with an IC_50_ value of 1322.6 ± 71.61 µg/ml (Figure 5), than in cancer cells. Furthermore, the antiproliferative effect of HE was confirmed through a colony formation assay (Figure 6), which indicated that HE exerts dose‐dependent inhibition of colony formation in PANC‐1 and MDA‐MB‐231 cells (Figure 6). A few studies have reported antiproliferative effects of banana extracts. Dahham et al. (2015) found that HE of a Malaysian variety of banana fruit flesh had growth inhibitory effects on MCF‐7 estrogen receptor‐positive breast cancer cells. Kamal et al. (2019) have shown anticancer and radioprotective effects of banana peel extracts in‐vivo.

### 3.5 Hexane extract of banana fruit flesh induces apoptosis

**FIGURE 7:**
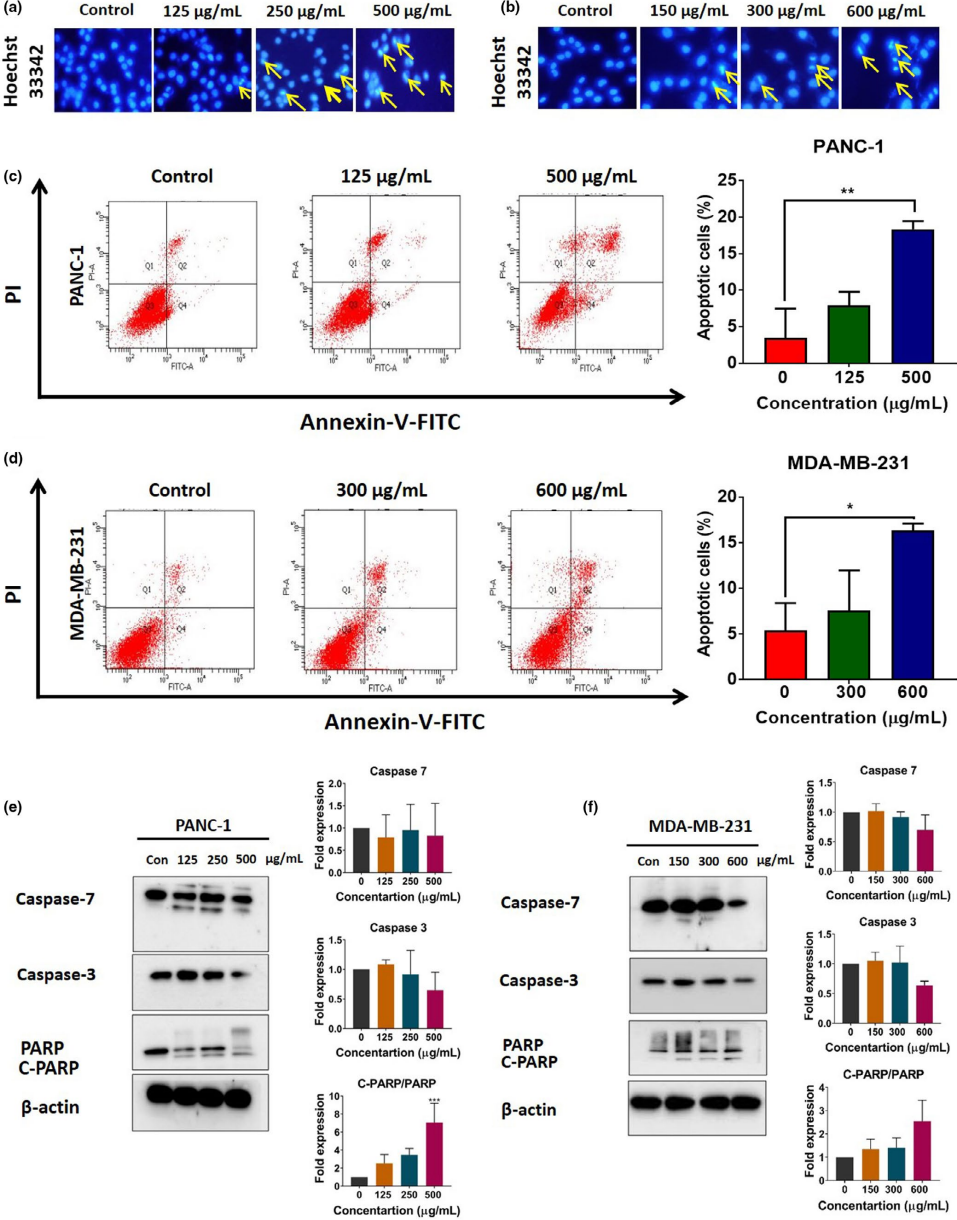
Hoechst 33342 staining of PANC‐1 (a) and MDA‐MB‐231 cells (b). Yellow arrows in Hoechst 33342‐stained cells indicate condensed chromatin. Representative flow cytometry plots obtained using Annexin V‐FITC/PI staining to assess apoptosis in PANC‐1 (c) and MDA‐MB‐231 cells (d) following exposure to HE for 48 h. Western blot analysis of the expression of apoptosis‐related proteins in PANC‐1 (e) and MDA‐MB‐231 cells (f) exposed to HE for 48 h. Representative bands for caspase 7, caspase 3, full PARP, and cleaved PARP (C‐PARP) are shown on the blots. Statistical comparison among groups was carried out using one‐way ANOVA with Dunnett's post hoc test. *p < .05 and **p < .01 compared to untreated cells (*n* = 3)

Evasion of apoptosis is a hallmark of cancer (Reed, 2000). Cellular apoptosis is associated with specific morphological and biochemical changes, including the formation of apoptotic bodies, cell shrinkage, blebbing of the plasma membrane, condensation of chromatin, and activation of caspases (Reed, 2000). To determine whether HE can mediate cytotoxic effects through induction of apoptosis, Hoechst 33342 staining was performed. As shown in Figure 7a,b, condensation of chromatin, a biochemical feature of apoptosis, was observed in PANC‐1 and MDA‐MB‐231 cells exposed to HE. Induction of apoptosis in response to HE was confirmed through annexin V/PI staining. HE‐treated PANC‐1 and MDA‐MB‐231 showed signs of early and late apoptosis in a concentration‐dependent manner. In PANC‐1 cells, the percentage of early and late apoptotic cells increased by 7.90% ± 1.86 and 18.30% ± 1.15 at the concentration of 125 and 500 μg/ml, respectively. The percentage of early and late apoptotic cells in MDA‐MB‐231 cells were 7.60% ± 4.43 and 16.30% ± 0.75 at the 300 and 600 μg/ml concentration of HE, respectively (Figure 7c,d). These results were supported by Western blot analysis of the relative levels of apoptosis‐related proteins in PANC‐1 and MDA‐MB‐231 cells exposed to HE. When treated with HE, reductions of caspase‐7 and caspase‐3 were observed in both cell lines, while the cleaved form of caspase‐7 was more abundant in PANC‐1 cells than in MDA‐MB‐231 cells. Moreover, HE dramatically increased the level of the cleaved form of poly(ADP‐ribose) polymerase (PARP), an apoptosis‐associated marker, in both PANC‐1 and MDA‐MB‐231 cells. In PANC‐1 cells, the level of the cleaved form of PARP increased by 7.04 ± 2.01 fold, whereas in MDA‐MB‐231 cells, the level of cleaved PARP increased by 2.55 ± 0.89 fold at the highest concentration (Figure 7e,f). Collectively, these results indicate that HE can induce apoptosis in PANC‐1 and MDA‐MB‐231 cells.

### 3.6 GC‐MS analysis of banana flesh hexane extract

HE was subjected to GC‐MS analysis to identify its lipophilic constituents. GC‐MS analysis of HE allowed tentative identification of several lipophilic compounds (Table 1). HE was rich in fatty acids, fatty acid esters, and several sterols, some of which have been tested previously for anticancer effects. For example, palmitic acid has been reported to exhibit selective anticancer effects in human leukemia cells (Harada et al., 2002). In addition, linoleic acid has been reported to possess a wide range of anticancer effects (Jóźwiak et al., 2020). A recent study demonstrated that oleic acid has anticancer effects based on the reduction of autophagy (Giulitti et al., 2021). Vilela et al. (2014) reported that fruits of several *Musa* species contain a noticeable amount of campesterol, stigmasterol, and beta‐sitosterol, justifying our GS/MS results. Furthermore, gamma‐sitosterol has been reported to induce apoptosis and cell cycle arrest in breast and lung cancer cells (Sundarraj et al., 2012). Recently, stigmasterol was found to induce apoptosis and autophagy through inhibition of the Akt/mTOR signaling pathway in gastric cancer cells (Zhao et al., 2021). Thus, based on the GC‐MS profile, our hypothesis is that the anticancer activity of HE extract might be caused by the several lipophilic compounds, including previously reported anticancer compounds, with synergistic anticancer properties (Giulitti et al., 2021; Harada et al., 2002; Jóźwiak et al., 2020; Sundarraj et al., 2012; Zhao et al., 2021).

## 4 CONCLUSION

The HE of banana flesh had the highest total polyphenol content (2.54 ± 0.60 mg GAE/g) and total flavonoid content (1.69 ± 0.34 mg RE/g), and showed strong free radical scavenging effects among the four kinds of banana flesh extracts. We report for the first time that banana flesh extract can exhibit cytotoxic and apoptotic effects in human pancreatic cancer PANC‐1 and human triple‐negative breast cancer MDA‐MB‐231 cells. Gas chromatography mass spectroscopy (GC‐MS) analysis revealed the presence of compounds that have been reported to have anticancer efficacy. Our findings suggest that banana flesh can be used as a dietary supplement for protection against pancreatic and breast cancers.

## CONFLICT OF INTEREST

The authors declare no conflict of interest.

## ETHICAL APPROVAL

The authors declare that this study did not involve human or animal subjects, and human and animal testing is unnecessary in our study.

## Supporting information

Figure S1

## Data Availability

The datasets generated and analyzed during the current study are available from the corresponding author upon reasonable request.
