# Supplementary material for: Antioxidant activity of banana flesh and antiproliferative effect on breast and pancreatic cancer cells
Source: Food Sci Nutr. 2022 Jan 26;10(3):740–50. doi: 10.1002/fsn3.2702 (PMC8907754; doi:10.1002/fsn3.2702)

**Figure S1.** Cytotoxic effects of banana flesh extracts in RAW 264.7 cells as assessed by the MTT assay (n=3). hexane extract (HE), chloroform fraction (CF), and ethanol fraction (EF), total ethanol extract (TE).


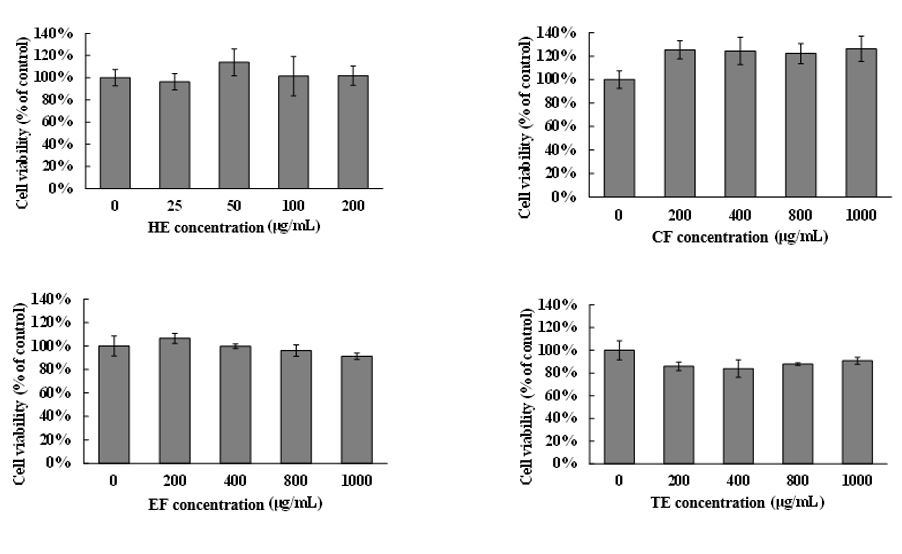

Supplement: Supplementary file 1 — Figure S1 [file FSN3-10-740-s001.docx]
